# Analysis of 31 STR loci in the genetic isolate of Carloforte (Sardinia, Italy)

**DOI:** 10.1590/S1415-47572009005000057

**Published:** 2009-09-01

**Authors:** Renato Robledo, Ignazio Piras, William Beggs, Carla Calò

**Affiliations:** 1 Dipartimento di Scienze e Tecnologie Biomediche, Università di Cagliari, Monserrato, CagliariItaly; 2 Dipartimento di Biologia Sperimentale, Università di Cagliari, Monserrato, CagliariItaly; 3 Department of Molecular Biology, Coriell Institute for Medical Research, Camden, New JerseyU.S.A

**Keywords:** autosomal STRs, population data, genotyping, Carloforte, Sardinia

## Abstract

The genotypes of 31 autosomal short tandem repeat loci in the population of Carloforte were analyzed, these representing a linguistic and genetic isolate located on the island of Sardinia (Italy). The markers span the entire length of chromosomes 19, 20, 21 and 22. Allele frequencies and statistical parameters were presented for all loci. Observed heterozygosity ranged from 0.279 to 0.884, and polymorphism information content from 0.552 to 0.886. All but two loci showed Hardy-Weinberg equilibrium after Bonferroni correction. The 31 short tandem repeat loci examined in the present work provide additional data on the genetic structure of the Carloforte population.

Carloforte is the only village located on the small island of San Pietro, off the southwestern coast of Sardinia. The history of the Carloforte population dates back to the 16^th^ century, when a number of families emigrated from Pegli, a small village in Liguria (Italy), now part of the city of Genova, to Tabarka, an island off the coast of Tunisia. During their time in Tabarka, a period of about two centuries, the Pegli community developed a successful business in tuna and coral fishing. At the beginning of the 18^th^ century, due to a decline in business and worsening relationship with the Bey of Tunis and Algiers, the community migrated to Sardinia and settled on the deserted island of San Pietro, where they founded Carloforte in 1738 (Vallebona, 1974). For about 10 generations, these Genovese migrants had very little contact with the mainland populations of both Tunisia and Sardinia, maintaining a separate cultural as well as genetic identity. The cultural aspect is evident in the Pegli dialect, which is still spoken nowadays, making the Carloforte population a linguistic isolate (Vona *et al.*, 1996). Earlier studies based on matrimonial structure, classical genetic markers and the incidence of a specific disease provided evidence that Carloforte is also a genetic isolate (Vona *et al.*., 1996; Heath *et al.*, 2001). In this paper, we present further data on the genetic structure of the Carloforte population by reporting on the distribution of 31 micro-satellite DNA loci. The markers employed were CA di-nucleotide repeats clustered in four linkage groups, representing chromosomes 19, 20, 21 and 22.

Historical documents list the names of the first settlers. Individuals selected for the present study were proven to be descendants of the village founders. Moreover, the participants were chosen for not having ancestors in common, at least up to the grandparental generation. Both criteria were ascertained by a complete genealogical search through the detailed family information available from City Hall records (Siniscalco *et al.*, 1999; Heath *et al.*, 2001).

About 10 mL of peripheral blood were collected from 50 voluntary participants, in vacuum tubes containing EDTA as anticoagulant. Permanent cultures of EBV-transformed B-lymphocytes were prepared and stored anonymously in the Coriell Repository. DNA was extracted by standard laboratory procedures. The study was approved by local ethics committees and all voluntary participants read and signed an informed consent form, in accordance with Declaration of Helsinki guidelines.

Individuals were genotyped using the ABI Prism Linkage Mapping Panel, version 2. The markers were amplified as single reactions and products pooled for analysis. About 50 ng of genomic DNA were amplified in a final PCR volume of 15 μL containing 1.5 μL 10x GeneAmp PCR buffer II, 1.5 μL 25 mM MgCl_2_, 1.5 μL 2.5 mM dNTP mix, 1.0 μL Primer Mix and 0.12 μL AmpliTaq Gold DNA Polymerase, with an ABI GeneAmp PCR System 9700 Thermal Cycler. PCR reactions were amplified by using the following PCR conditions: a 12 min hold at 95 °C was followed by 10 cycles of 15 s at 94 °C, 15 s at 55 °C, 30 s at 72 °C and 20 additional cycles of 15 s at 89 °C, 15 s at 55 °C and 30 s at 72 °C, with a final extension of 10 min at 72 °C.

The amplified PCR products were pooled at a ratio of 5 μL of each FAM labeled product and 10 μL of each HEX and NED labeled one. D.I. water was added to a final volume of 100 ul. The pooled PCR product was mixed with loading buffer at a 2:3 ratio for a 5 μL final volume (loading buffer contained 4.5 μL formamide, 1.0 μL blue dextran and 1.5 μL of S400HD-ROX as an internal size standard). The mixture was heated at 95 °C for 3 min and cooled on ice for 3 min, before being separated on an ABI 377 Sequencer, using a 36 cm denaturing polyacrylamide gel for resolution of di-nucleotide repeat products. Separated allele fragments were analyzed using ABI GeneScan and Genotyper software and genotypes scored.

Allele frequencies were estimated by gene counting. Both observed and expected heterozygosity were calculated with the GENETIX program (v. 4.0). Deviation from Hardy-Weinberg equilibrium (HWE) was tested by the Markov chain, using the GENEPOP program (v. 4.0). Statistical parameters of population genetics and forensic interest, namely polymorphism information content (PIC), power of discrimination (PD), and power of exclusion (PE), were calculated using Power Stats package v. 1.2 (Promega Corporation, USA).

To verify whether the population had incurred a recent bottleneck, data were analysed using the BOTTLENECK program, v 1.2 (Cornuet and Luikart, 1996). This program allows for the evaluation of observed and expected heterozygosity, and determines statistical difference based on equilibrium mutation/genetic drift. This was obtained through simulating the coalescent process of *n* genes under the two-phase mutation model (TPM), which was proved to better fit micro-satellite analysis (Di Rienzo *et al.*, 1994). The TPM mainly consists of one-step mutations, with a small percentage (5%-10%) of multi-step changes.

Allelic frequencies of the 31 STR loci tested in the population of Carloforte are reported in Table S1. Hardy-Weinberg equilibrium (HWE) and population parameters are shown in Table 1.

The 31 loci showed a high degree of heterozygosity, varying from 27.9% for marker D21S1256 to 88.4% for marker D22S283. The exact test based on Markov chain analysis revealed that 6 out of 31 markers did not meet Hardy-Weinberg equilibrium, whereas after Bonferroni correction, only two of these, D20S173 and D21S1256, showed an excess of observed homozygotes. The high degree of isolation of the Carloforte population, together with the reported deviation from Hardy Weinberg expectation, prompted us to verify the possibility of a recent bottleneck in Carloforte through the BOTTLENECK program. Under the assumption of the two-phase model of mutation (TMP), the Wilcoxon test revealed non-significant heterozygosity (p = 0.459), thereby indicating that, following initial colonization events, there were no further contractions in the size of the Carloforte population, this being in agreement with historical records. Moreover, the mode shape test showed an L-shaped distribution, which is also consistent with the absence of a recent bottleneck (data not shown).

The high level of heterozygosity in a genetic isolate like Carloforte, with a long history of endogamous and consanguineous marriages, may at first be surprising. We believe that the sampling strategy accounts for the findings: differences among individuals were maximized by selecting participants with no ancestor in common, at least up to the grandparental generation. This could be very important in association studies, where it is necessary to analyze large numbers of individuals in order to detect statistically significant deviations in allelic distribution between cases and matched controls. In a previous study, on applying the sampling strategy as described above, we genotyped 55 individuals from the Carloforte population at 5 unlinked micro-satellite loci in order to obtain an accurate description of their genomic profile. We showed that allele frequencies at all loci were practically the same down to a subset of 20 individuals (Siniscalco *et al.*, 1999). Therefore, once a breeding unit is identified through the recording of marriage patterns over the last five generations, a small sampling of contemporary descendants is still representative of its founder group, provided that the individuals selected had their ancestors all born in the same breeding unit, but unrelated for, at least, the last two generations. An obvious consequence of the proposed sampling strategy is a reduction in the cost of association studies, due to a lower number of individuals having to be genotyped.

Finally, Table 2 shows parameters of forensic interest. The combined power of both discrimination and exclusion were absolutely discriminating, their values being, respectively, 7.7 x 10^-33^ and 0.999999999918. It has been reported that genetic isolation could reduce the capacity for paternity exclusion, without significantly affecting the power of discrimination (de Pancorbo *et al.*, 2000). This could be a critical issue in forensics, for example when setting up a database from a small village or population. In our case, the power of exclusion was still very high, again probably as a result of the sampling strategy. The combined matching probability for the 31 STRs was 0.9999999999923. Therefore, the 31 STR loci examined in the present work, specifically designed for population genetics studies, also turned out to be suitable for general forensic applications.

## Supplementary Material

The following online material is available for this article:

This material is available as part of the online article from http://www.scielo.br/gmb.

Table S1 Allele frequencies of the 31 STR loci in the population of Carloforte.

## Figures and Tables

**Table 1:** Heterozygosity, number of alleles detected and HWE evaluation (indicated as probability values) of 31 investigated markers in the Carloforte population.

Locus	Observed heteroz.	Expected heteroz.	N. of alleles	HWE
D19S216	0.619	0.671	8	0.722
D19S884	0.683	0.867	10	0.020
D19S226	0.829	0.828	14	0.909
D19S414	0.750	0.759	8	0.551
D19S220	0.750	0.798	10	0.197
D19S420	0.727	0.741	9	0.154
D19S902	0.810	0.759	8	0.865
D19S571	0.738	0.798	8	0.009
D19S418	0.727	0.697	7	0.124
D19S210	0.641	0.707	5	0.149
D20S889	0.769	0.825	14	0.742
D20S115	0.614	0.646	4	0.249
D20S186	0.864	0.840	10	0.325
D20S112	0.714	0.753	11	0.338
D20S195	0.725	0.843	11	0.116
D20S107	0.841	0.829	9	0.840
D20S119	0.698	0.764	6	0.337
D20S178	0.878	0.851	8	0.862
D20S196	0.744	0.819	10	0.622
D20S100	0.698	0.768	10	0.220
D20S173	0.316	0.581	6	0.000
D21S1256	0.279	0.736	7	0.000
D21S1914	0.618	0.865	8	0.001
D21S1252	0.864	0.842	8	0.568
D21S266	0.698	0.727	10	0.720
D22S420	0.590	0.733	7	0.069
D22S315	0.738	0.743	10	0.943
D22S280	0.837	0.800	8	0.308
D22S283	0.884	0.895	11	0.828
D22S423	0.864	0.823	11	0.705
D22S274	0.860	0.773	7	0.125

**Table 2:** Statistical relevant data for 31 investigated markers in the Carloforte population. MP: matching probability, PD: power of discrimination, PIC: polymorphism information content, PE: power of exclusion.

Locus	MP	PD	PIC	PE
D19S216	0.162	0.838	0.616	0.314
D19S884	0.049	0.951	0.852	0.402
D19S226	0.053	0.947	0.810	0.654
D19S414	0.112	0.888	0.723	0.510
D19S220	0.081	0.919	0.774	0.510
D19S420	0.120	0.880	0.714	0.472
D19S902	0.092	0.908	0.735	0.617
D19S571	0.099	0.901	0.768	0.490
D19S418	0.146	0.854	0.669	0.472
D19S210	0.143	0.857	0.666	0.343
D20S889	0.055	0.945	0.809	0.543
D20S115	0.204	0.796	0.577	0.308
D20S186	0.069	0.931	0.820	0.722
D20S112	0.101	0.899	0.724	0.451
D20S195	0.060	0.940	0.826	0.468
D20S107	0.063	0.937	0.806	0.677
D20S119	0.100	0.900	0.726	0.425
D20S178	0.055	0.945	0.833	0.751
D20S196	0.062	0.938	0.795	0.500
D20S100	0.094	0.906	0.740	0.425
D20S173	0.259	0.741	0.552	0.070
D21S1256	0.158	0.842	0.692	0.055
D21S1914	0.057	0.943	0.849	0.313
D21S1252	0.062	0.938	0.822	0.722
D21S266	0.095	0.905	0.705	0.425
D22S420	0.122	0.878	0.690	0.279
D22S315	0.095	0.905	0.714	0.490
D22S280	0.097	0.903	0.773	0.670
D22S283	0.035	0.965	0.886	0.762
D22S423	0.063	0.937	0.801	0.722
D22S274	0.120	0.880	0.739	0.716
