# Supplementary material for: Analysis of 31 STR loci in the genetic isolate of Carloforte (Sardinia, Italy)
Source: Genet Mol Biol. 2009 Sep 1;32(3):462–5. doi: 10.1590/S1415-47572009005000057 (PMC3036058; doi:10.1590/S1415-47572009005000057)
Supplement: Table S1 — Allele frequencies of the 31 STR loci in the population of Carloforte. [file gmb-32-3-462-suppl1.pdf]

Table S1 - Allele frequencies of the 31 STR loci in the population of Carloforte. Alleles are reported on the basis of their fragment length.

Locus	Frequency
D19S216	
258	0.012
260	0.095
262	0.012
266	0.452
268	0.060
270	0.024
272	0.333
274	0.012
D19S884	
95	0.085
99	0.049
101	0.183
103	0.073
105	0.037
107	0.134
109	0.073
111	0.195
113	0.024
115	0.146
D19S226	
237	0.024
239	0.012
241	0.317
243	0.061
245	0.037
247	0.195
249	0.012
251	0.122
253	0.098
255	0.037
257	0.037
259	0.012
261	0.024
265	0.012
D19S414	
170	0.341
172	0.011
184	0.046
186	0.102
188	0.125
190	0.307
192	0.046
194	0.023
D19S220	
268	0.046
272	0.011
274	0.034
278	0.023
280	0.341
282	0.227
284	0.102
286	0.114
288	0.023
290	0.080
D19S420	
94	0.023
98	0.023
102	0.046
104	0.057
108	0.171
110	0.443
112	0.136
114	0.091
120	0.011
D19S902	
241	0.119
245	0.095
247	0.012
251	0.429
253	0.119
255	0.119
257	0.060
259	0.048
D19S571	
288	0.202
308	0.012
310	0.179
312	0.214
314	0.274
316	0.083
318	0.024
324	0.012
D19S418	
90	0.091
92	0.034
94	0.500
96	0.102
98	0.148
100	0.102
104	0.023
D19S210	

179	0.115
181	0.244
183	0.449
187	0.090
189	0.103
D20S889	
93	0.026
95	0.180
97	0.026
99	0.013
101	0.333
103	0.090
105	0.026
107	0.051
109	0.115
111	0.039
113	0.026
115	0.013
117	0.051
119	0.013
D20S115	
238	0.011
240	0.261
242	0.466
244	0.261
D20S186	
119	0.136
125	0.125
127	0.011
129	0.034
131	0.216
133	0.023
135	0.239
137	0.125
139	0.023
141	0.068
D20S112	
211	0.048
215	0.095
219	0.024
223	0.024
225	0.417
227	0.191
229	0.155
231	0.012
233	0.012
235	0.012
237	0.012
D20S195	
131	0.038
139	0.188
141	0.025
143	0.050
145	0.263
147	0.075
149	0.163
151	0.013
153	0.075
155	0.100
161	0.013
D20S107	
202	0.011
208	0.193
210	0.034
212	0.205
214	0.125
216	0.239
218	0.125
220	0.046
222	0.023
D20S119	
109	0.012
113	0.163
115	0.058
117	0.256
121	0.186
123	0.326
D20S178	
182	0.134
184	0.110
186	0.183
188	0.049
190	0.195
192	0.183
194	0.098
196	0.049
D20S196	
265	0.198
273	0.012
277	0.023
279	0.035
285	0.047
287	0.151
289	0.279

291	0.186
293	0.047
295	0.023
D20S100	
217	0.035
219	0.012
223	0.198
225	0.395
227	0.151
229	0.012
231	0.081
233	0.023
235	0.070
239	0.023
D20S173	
129	0.145
173	0.079
175	0.066
177	0.618
179	0.066
183	0.026
D21S1256	
107	0.163
109	0.349
111	0.093
113	0.023
115	0.326
117	0.023
121	0.023
D21S1914	
264	0.103
266	0.132
268	0.132
270	0.177
272	0.162
274	0.147
276	0.074
278	0.074
D21S1252	
148	0.011
150	0.171
156	0.205
158	0.182
162	0.046
164	0.148
166	0.091
168	0.148
D21S266	
159	0.163
161	0.081
163	0.477
165	0.047
167	0.035
171	0.035
175	0.047
177	0.047
179	0.012
181	0.058
D22S420	
154	0.103
156	0.013
158	0.333
160	0.346
162	0.154
164	0.026
168	0.026
D22S315	
178	0.024
184	0.024
186	0.012
190	0.012
196	0.083
198	0.095
200	0.429
202	0.226
204	0.048
206	0.048
D22S280	
214	0.105
216	0.163
218	0.105
220	0.221
222	0.314
224	0.023
226	0.058
228	0.012
D22S283	
139	0.105
141	0.105
143	0.151
145	0.128
147	0.047
149	0.116
151	0.081
153	0.070
155	0.105

157	0.070
161	0.023
D22S423	
288	0.023
294	0.023
296	0.273
298	0.148
300	0.023
302	0.080
304	0.057
306	0.034
308	0.250
310	0.080
316	0.011
D22S274	
284	0.047
286	0.291
288	0.302
290	0.035
292	0.174
294	0.128
296	0.023
